# Promoting good and equal health by advancing vulnerable students’ employment and income opportunities: Protocol for a multiple method research program leveraging student and school staff perspectives

**DOI:** 10.1371/journal.pone.0360645

**Published:** 2026-10-13

**Authors:** Tina M. Olsson, Hanna Ginner Hau, Sabina Kapetanovic, Diana Kajic, Therése Skoog

**Affiliations:** 1 Department of Social Work, School of Health and Welfare, Jönköping University, Jönköping Sweden; 2 Department of Special Education, Stockholm University, Stockholm, Sweden; 3 Department of Psychology, Education, and Sociology, University West, Trollhättan, Sweden; 4 Department of Psychology, Gothenburg University, Gothenburg, Sweden; University of Coimbra: Universidade de Coimbra, PORTUGAL

## Abstract

School-level social determinants of health (SDOH) account for a large share of variation in students’ health behaviors. Because educational attainment is itself a key social determinant of health—shaping later employment, income, and health—efforts to promote equitable health must include understanding and improving school climate as a mechanism influencing academic achievement and wellbeing. This project examines how school-level SDOH develop and how they relate to later employment, income, and health outcomes in a representative sample of Swedish young people. It also investigates how school staff and students perceive the school’s ability to address school-level SDOH and develops and pilots an intervention aimed at strengthening these determinants. This multi-method, multi-informant study uses a large longitudinal dataset following a representative cohort from ages 12–20. Additional primary data will be collected from school staff and students to assess perceptions of school-level SDOH. The project will also design and test an intervention intended to improve students’ academic achievement, a central pathway influencing later employment, income, and health. Combining longitudinal data with new qualitative and quantitative materials allows the project to examine the development of school-level SDOH and the mechanisms through which they shape long-term outcomes. Education is essential for strengthening skills, economic opportunities, and health equity. Strong links exist between education level, employment, income, and health, making this project relevant for students, families, school personnel, and broader policy development. Findings may guide schools and policymakers in improving school environments to support achievement and long-term health equity. The project involves extensive data collection and collaboration across disciplines and universities. By integrating multiple methods, it aims to advance knowledge on how education influences employment, income, and health while informing school-level and policy-level strategies to support academic achievement and wellbeing.

## Introduction

A recent longitudinal cohort study of more than 350,000 adults revealed that environmental (e.g., lifestyle) factors are independently associated with lifespan and that a healthy lifestyle attenuates the genetic risk of a short lifespan [1]. Sweden has committed itself to the principles held in the United Nations’ Universal Declaration of Human Rights [2] and the World Health Organization statutes [3], which express the same thing – everyone should have equal opportunities for good health and a long life. In reality, not all people have the same opportunities for good health. There are significant differences in health, risk of illness, and expected lifespan between people in different professions, with varying levels of education, or with different incomes. These differences can be seen at the community level in Sweden [4]. Differences in these conditions can be identified early in life. Children with different family backgrounds have different prerequisites for good health, just as their parents and other adults have different opportunities for good health [5]. As health disparities and vulnerabilities often take root in childhood and persist over time, addressing these must begin in childhood.

Children spend a considerable amount of time in school, and as such, schools are an important arena for shaping children’s knowledge, attitudes, behaviors, and outcomes. School-level social determinants of health (SDOH) can be understood as the modifiable social, organizational, and environmental characteristics of schools that influence students’ health and developmental outcomes [6]. School-level SDOH (e.g., school climate, student socioeconomic status, school fitness programs) have been found to explain up to 40% of the variation in students’ health behaviors [6], and educational attainment itself is a primary social determinant of health which may work through one of several pathways [7] including a pathway via employment and income. Children who enroll in poor-quality schools with fewer resources, more violence, and a distressed school climate, however, are less likely to complete school and more likely to follow a path toward worsened physical and mental health and fewer opportunities in relation to employment and income [8,9]. This, combined with increased school segregation in Sweden [5], may mean that the school environment is becoming an arena for increasing disparity instead of decreasing disparity.

Within the school-level social determinants of health framework, school climate is one of the most proximal and potentially malleable determinants experienced by students. School climate, as a critical feature of students’ everyday environment, is understood as a multidimensional feature of school life, encompassing five domains: students’ and staff members’ perceptions of safety, teaching and learning, interpersonal relationships, supportive leadership resources, and the broader quality of the school environment [10]. Indeed, previous studies have shown that both teacher and student ratings of school climate are significantly associated with school achievement [11,12]. However, there are serious concerns about the climate in schools in Sweden and internationally. In national surveys of exposure to crime in Sweden, students report the school environment as the most common location for both minor and more severe assaults, as well as for physical sexual harassment among both girls and boys [13]. Since 2015, the proportion of those reporting exposure to minor assault in the school environment (over the past 12 months) has increased, among both boys (from 58 percent to 68 percent) and girls (from 38 percent to 50 percent). In addition, 5% of boys and 9% of girls report that they have stayed home from school due to fear or worry about being threatened, harassed, or exposed to violence or crime at school [14]. Therefore, in attempts to achieve good and equal health for all, the development of healthy schools must include attempts to understand and impact school climate as an important mechanism for school achievement and student health, which together benefit students’ later employment, income opportunities, and health outcomes.

During the past several generations, education has become the principal pathway to financial security, stable employment, and social success [15]. At the same time, youth have experienced increasingly unequal educational opportunities that depend on the schools they attend, the neighborhoods they live in, and the financial resources of their family [5]. Approximately 15% of students in Sweden are not qualified to attend high school as they have not achieved the necessary grades during middle school [16]. For students with low grades upon entry into middle school, the chance of improving grades by the 9th grade is not better than 50/50 for certain subjects. Whether or not a student completes the 9th grade or high school has a profound impact on their later income opportunities [17]. Despite tremendous prior research, critical questions about the education-health relationship remain unanswered, partly because education and health are intertwined over the lifespan within and across generations and are inextricably embedded within the broader social context. In addition, and nearly universally, the education-health literature operationalizes education in terms of attainment, as years of schooling, or as completed education. Attainment, however, is only the endpoint of an extended and extensive formal schooling process, where relationships with peers, teachers, and other factors within the school context may shape schooling trajectories (e.g., attainment) and health [18]. This has led to calls for the urgent need for new directions in education-health research that can [1] capture education in action as it generates and/or constrains opportunity during childhood in current cohorts, [2] expand the operationalization of education beyond attainment to the consideration of the educational processes that precede attainment and their impact on health, and [3] reconceptualize education as not only a vehicle for social success, valuable resources, and good health, but also as an institution that reproduces inequality across generations [19].

Longitudinal research demonstrates that children facing various hurdles in school (e.g., behavioral, social, emotional) often struggle to achieve academic success [20]. Moreover, they are less likely to pursue higher education, more prone to involvement in criminal activities, and they face elevated risks of health issues, substance abuse, depression, and other mental health challenges [21]. Conversely, children who possess strong social skills tend to exhibit higher levels of engagement in school and foster positive relationships with their peers [22,23]. These “non-cognitive” skills (e.g., relationship, behavior) are increasingly recognized as essential for lifelong success in both the labor market [24] and for health [25,26].

Research underscores the significant role of non-cognitive abilities in shaping educational achievements across different social strata, contributing to the perpetuation of social inequalities during schooling [26]. Additionally, these skills serve as potent predictors of adult outcomes, including involvement in criminal behavior, health status, and access to higher education [27–29]. Such findings underscore the profound impact of non-cognitive skills on employment prospects, income potential, and overall health and well-being.

A recent systematic review of the international literature on schoolwide interventions to impact student and teacher perspectives on school climate found only 3 studies (of 3,859) that were of sufficient methodological quality to draw conclusions [30] and only two studies could be considered methodologically rigorous [31–33]. None of the included studies were conducted in Sweden. Investigators concluded that it is reasonable that school climate can be altered by school-based interventions. The strongest empirical support and evidence of effectiveness was found in interventions designed to focus on Social and Emotional Learning and School-Wide Positive Behavioral Interventions and Supports [31,32]. Investigators highlighted that it is premature to conclude that any interventions are ineffective due to low methodological quality and few replications and, as such, there is still much to learn about interventions to impact school climate (i.e., non-cognitive skills). As such, additional high-quality research on school-based intervention is needed to move science, policy, and practice forward. Understanding the role of the schooling process in health outcomes is relevant for science as it can help us better understand this complex process. In addition, understanding the role of the schooling process in health outcomes is relevant for policy as it can uncover whether interventions should be aimed at increasing educational achievement (e.g., attainment directly), whether targeting other processes within the school context is important for educational achievement (e.g., school climate), or whether targeting should be multimodal and multilevel [34].

Most studies have treated educational attainment as a starting point, a driver of opportunities in adulthood. However, the educational context also functions to reproduce inequality. The explicit recognition of the dual function of education is critical to developing our understanding of the education-health trajectory and educational policies that would avoid unintended consequences of increasing inequality. Increasing our understanding of long-term educational processes, and conceptualizing education as a vehicle for social success as well as an institution that may reproduce inequality across the education health trajectory, would increase our understanding of how the school context impacts health disparities and wellbeing [35].

### Theoretical background

This project has been developed based on the current literature and research practice recommendations in the field of prevention science [36–40] and rests solidly on two specific theoretical frameworks. The first framework is the social determinants of health framework (SDOH [41],), which focuses on the social, economic, and environmental factors influencing health outcomes. Within this framework schools are critical social determinants of health [42]. Accordingly, schools are not only sites of academic instruction, but also social contexts in which young people encounter relationships, norms, resources, risks, and opportunities that may shape both educational and health-related trajectories. In line with this perspective, the present project focuses on school climate as a central and modifiable school-level determinant of health (see hypothetical model in Fig 1). Within this school-based social determinants framework, early childhood vulnerabilities are expected to influence outcomes through both adverse and promotive mechanisms. Adverse mechanisms include peer difficulties, exposure to violence, substance use, and other risk processes that may undermine academic achievement and wellbeing. Promotive mechanisms include socioemotional skills, resilience, supportive relationships, school belonging, and prosocial behavior. These mechanisms may shape students’ academic achievement and later educational attainment, while also contributing directly or indirectly to mental health and wellbeing. School climate is hypothesized to moderate these pathways by reducing the impact of adverse mechanisms and enhancing the influence of promotive mechanisms on educational and health outcomes.

**Fig 1:**
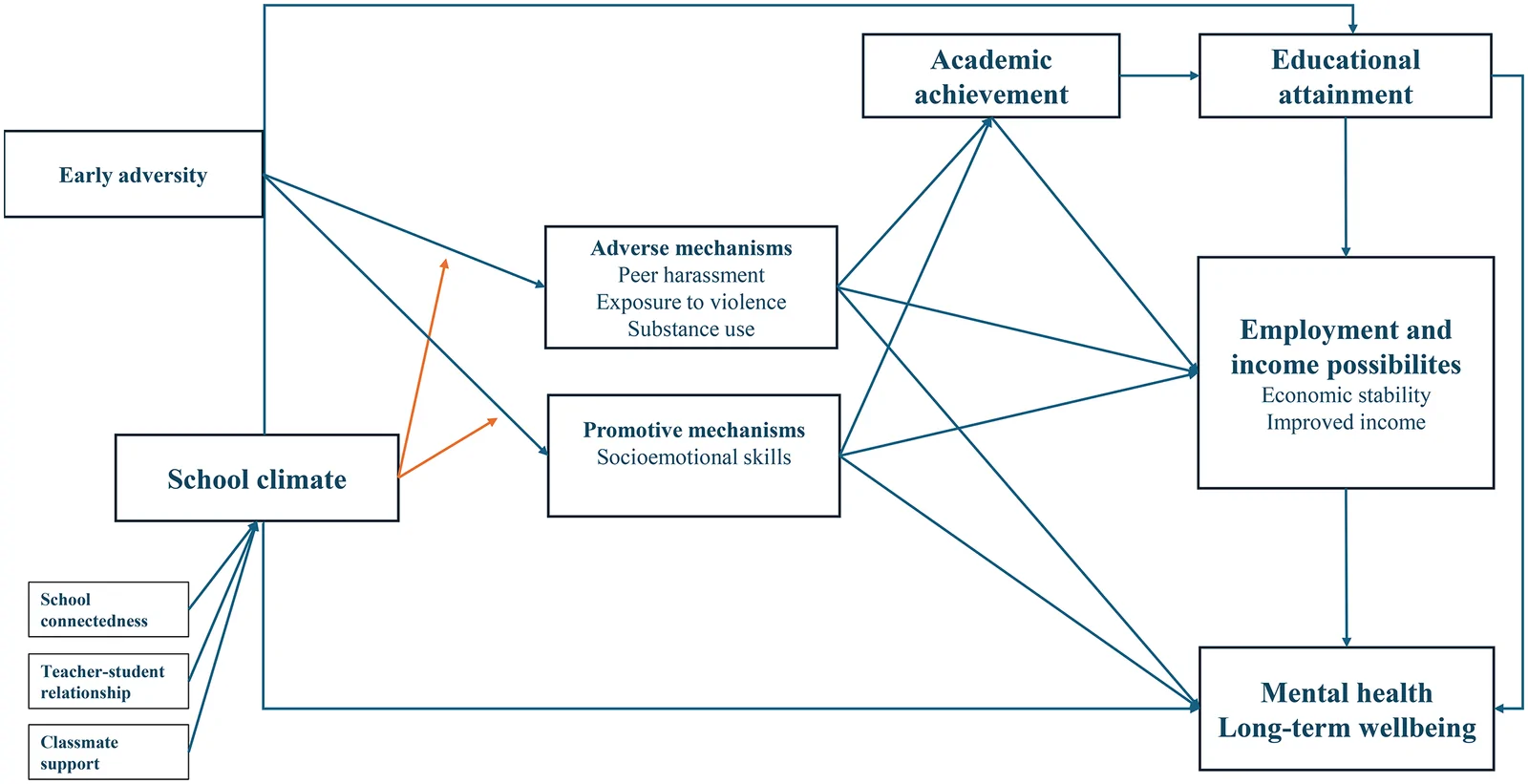
Conceptual model of school climate as a social determinant of health across the life course.

The second framework, the life-course perspective [43], complements the SDOH framework by explaining how school-based social determinants of health may matter over time. While the school-level SDOH framework identifies the school context as an important setting for health and educational development [42], the life-course perspective clarifies how experiences within this setting may accumulate and influence later trajectories. School climate may affect students’ academic achievement, socioemotional skills, resilience, health behaviors, and mental wellbeing during childhood and adolescence. These early outcomes may then shape educational attainment, employment opportunities, income, economic stability, and health in later life. Thus, the life-course perspective provides the temporal logic linking school climate to both immediate and longer-term outcomes. Thus, the project examines school climate not as an isolated school characteristic, but as a potentially important starting point in a broader pathway linking school experiences to later educational and health outcomes.

### Study purpose and research questions

The purpose of this project is to investigate the school context as an arena for promoting good and equitable health in the short and long term. The project focuses on two central aspects of school climate in particular: interpersonal relationships, including peer relationships and teacher-student relationships, and institutional environment, encompassing physical surroundings and students’ sense of belonging and attachment to school. We examine how these aspects of school climate are associated with school achievement and with later employment, income opportunities, and health outcomes.

***Research question 1***: How does school climate moderate the relationship between early vulnerability (e.g., socioeconomic disadvantage, family instability) and academic achievement (e.g., eligibility for upper secondary school)?

*Hypothesis 1*: School climate moderates the association between early vulnerability and short-term academic outcomes, such that the negative relationship between early vulnerability and eligibility for upper secondary school is weaker in schools with a more positive climate.

*Hypothesis 2*: School climate moderates the association between early vulnerability and early-adult outcomes, such that the likelihood of not being in education, emplyment or training (NEET) is less strongly predicted by early vulnerability among students attending schools with a more positive climate.

*Hypothesis 3*: School climate moderates the association between early vulnerability and longer-term educational attainment, such that early vulnerability is less strongly associated with lower educational completion in schools with more positive climates.

***Research question 2***: How does school climate moderate the relationship between early vulnerability and later employment, income, and health?

*Hypothesis 4*: School climate moderates the association between early vulnerability and later employment, such that early vulnerability is less strongly associated with unemployment or unstable employment among students who attended schools with a more positive school climate.

*Hypothesis 5*: School climate moderates the association between early vulnerability and later income, such that the negative relationship between early vulnerability and adult earnings is weaker for individuals who experienced a more positive school climate.

*Hypothesis 6*: School climate moderates the association between early vulnerability and later health outcomes, such that early vulnerability is less strongly associated with poorer physical and mental health in adulthood among those who attended schools with a more positive school climate.

The following research questions are hypothesis generating:

***Research question 3***: What resources (e.g., economic resources, internal- and external support, competencies) do school staff (e.g., teachers, leadership, and student health) experience as present or lacking in efforts to work effectively with school-level social determinants of health?

***Research question 4:*** What resources (e.g., competence, opportunity, motivation) do students experience as present or lacking within the school context for promoting school-level social determinants of health?

***Research question 5***: Based on the results of RQ1-RQ4, how might a school-based intervention be designed to improve school climate and student health behaviors (e.g., pro-social behavior)?

***Research question 6***: How does the pilot intervention impact school climate and student health behaviors in a small-scale trial?

***Research question 7***: To what extent do students and teachers experience the pilot intervention as appropriate, acceptable, and feasible?

### Swedish legislative context

In Sweden, the educational landscape is shaped by comprehensive legislation (e.g., the School Act) designed to ensure a high standard of learning and a high minimum level of knowledge, skills, and abilities for all students. However, this well-intentioned system is designed in a way which leads to the exclusion a group of students from further education (i.e., entering senior high school). When students fail to meet the requirements set by compulsory school subjects, they are not allowed to enter high school. The legislation mandates that students must pass requirements in at least 8 subjects (including Swedish, English, and Mathematics) when leaving compulsory school (i.e., grade 9). Those who fall short of these standards find their pathways to further education restricted, as entry to upper secondary school requires a certain level of achievement. In Sweden, senior high school education is key to stable employment and good income opportunities [44]. Consequently, current Swedish school legislation limits opportunities for students who fail to pass at least 8 compulsory school subjects (typically students who are also vulnerable in other regards), potentially hindering their educational and career prospects, and in turn their employment and income opportunities. This project has the potential to strengthen our understanding of how this legislation impacts diverse students’ educational, employment and income opportunities, and health and what schools might do to boost these opportunities through targeted intervention.

### Project novelty

This project is novel in several ways. First, it will leverage a current longitudinal data set that combines self-reports and teacher reports on a range of school-level social determinants of health, such as student functioning in school and school climate. In addition, our data set includes measures of student-level characteristics and vulnerabilities that may impact equity, such as gender, ethnicity, function, and ability. Our data set also includes linked register data, which enables longer-term assessment of trajectories toward school achievement, income and employment, and health. Unlike prior research, this enables us to investigate school-level social determinants of health, their development and change over time within the school setting, and their impact on employment and health among a representative sample of Swedish students. Second, we will, in collaboration with partnership schools, investigate students’ and teachers’ perceived ability to work effectively with school-level social determinants of health something lacking in the extant literature. Finally, in collaboration with partnership schools, we will develop and test a pilot intervention to positively impact the school-level SDOHs identified through the study of RQ1-RQ4. As such, this project is multimethod and multi-informant which adds empirical power to the results that will be generated from this project.

## Materials and methods

### Ethics statement

Written ethical approval was provided for this study by the Swedish Ethical Review Authority (2025-05596-01, 2024-03620-02, 2021−01443).

### Study design, empirical material, data collection, and analysis

This multi-method and multi-informant project will leverage a large longitudinal data set that follows a representative sample of children from ages 12–20, collection of qualitative data from school representatives and students as well as development and pilot testing of an intervention designed to boost children’s perceived school climate.

### The Gothenland Millennium Cohort longitudinal data set

To answer RQ1 and RQ2 we will conduct analyses on data from the Gothenland Millennium Cohort (GMC [45],). With the prospective longitudinal design, GMC data has provided a basis for studies on transitions in adolescence by collecting information about adolescents’ mental health, school performance, school climate, and relationships with teachers and peers, as well as orientation toward norm breaking behaviors, such as violence and delinquency. These are studied by means of annual adolescent self-reports, teachers’ reports, and school registry data on participating students’ school performance. These registers provide valuable opportunities to study social, educational, and labor market related adaptation over time. As such, we have a unique longitudinal data set which allows us to study how school level factors influence later school achievement, employment and income outcomes, and health in a current Swedish sample.

#### Participants and procedure.

The adolescents in the GMC (n = 1885) were followed for up to six years, from age 12 or 13 to age 18, with comprehensive diagnostic interviews as the last step in the program. Students from special education schools as well as students following the adapted curriculum while integrated in regular schools were also included in the sample. The data collection started in 2013 with two cohorts from four municipalities in West Sweden where 6th and 7th grade students from all schools in these municipalities were invited to participate in the study. Their participation ended in 2020 when students were in their final year of high school. These cohorts are currently followed up to age 20 through various national data registers which will be harmonized as part of the Healthy Schools project.

#### Measures.

The following measures, using adolescent and to some extent parent and teacher self-reports, utilized across all the waves, will be used to address RQ1 and RQ2. Measures were selected to operationalize the key components of the conceptual model presented in Fig 1. Accordingly, the measures are organized into sections corresponding to (1) early childhood vulnerability, (2) school climate, (2) adverse mechanisms, (3) promotive mechanisms, (4) academic achievement, (5) mental health and wellbing, and (6) later educational, employment, and income outcomes. This structure reflects the hypothesized pathways through which school climate may influence developmental, educational, socioeconomic, and health trajectories across adolescence into adulthood.

Early childhood vulnerability is operationalized through the following measures:

***Socioeconomic disadvantage*** is measured via youth self-reports on four questions on perceived family economics. Questions such as “How would you rate your family’s economy compared with other families” and “How would you rate your family’s economy compared to other students in your class” are answered on a three-point semantic scale (e.g., less, about the same, more). Questions on socioeconomic disadvantage are also measured via parental self-reports with question such as, “Has the family been receiving social welfare grants during the past three years?”. In addition, family socioeconomic status is collected from national register data on personal income and finances.

***Parental unemployment*** is measured via national register data on employment records. Parental unemployment is also measured via parental self-reports on employment status.

***Family instability*** is measured via youth self-reports with question such as “Are either of your parents remarried or living with a new partner?”. In addition, family instability indicators are collected via national register data which includes data on changes in marital status, length of time in marital status, and family constellation over time.

***(Im)migrant background*** is measured via national registers on year of immigration to Sweden and place of birth for parents and children.

***Neighborhood disadvantage*** is measured via youth self-reports on the four-question neighborhood problem battery which (45; e.g., “Vandalism is common in my neighborhood”).

***Violence exposure and trauma*** is measured with the Childhood Trauma Questionnaire and response to an additional item, “I witnessed violence between adults in my home” which investigate experiences prior to age 12. In addition, four items from youth self-reports are used to assess traumatic experiences (e.g., “Have you experienced a serious event, accident, act of violence, or catastrophe that still upsets you?”)

***Disability and daily functioning*** are measured via youth self-reports. Questions include assessment of whether the respondent (1) has a specific disability and (2) the degree of difficulty they experience due to the identified disability. These questions are asked over four categories of disability: neuropsychiatric, cognitive, psychological, or somatic. Additionally, daily functioning is measured via a self-report battery which assesses how often specific activities are undertaken (e.g., “How often are you on time to school?”) and how important the youth perceives these activities to be (e.g., “How important to you think it is to come to school on time?”; 45).

School climate is operationalized through the following measures:

***School connectedness*** [46] assesses the extent to which students are satisfied with school and how they perceive their overall school experiences. The measure contains five items (e.g., How would you describe the relationship between you and school?) rated from 1 (the best/as best friends) to 5 (the worst/as enemies). Also, teachers have reported on their perceived student school satisfaction in wave 1–3.

***Relationship with teachers*** [46] assesses adolescents’ perceived teacher-student relationship with three items (e.g., “Do the teachers care about the students?”) rated from 1 (yes, all of them) to 4 (no, hardly anyone).

***Support from classmates*** [46] assesses adolescents’ perceptions of relational support from classmates using four items (e.g., “In my class, we help each other out”) rated from 1 (yes, completely true) to 4 (not true at all).

Adverse mechanisms are operationalized through the following measures:

***Cutting Class*** [46] is a single item used to measure this construct (i.e., “Have you cut class this semester?”) rated from 1 (no, has not happened) to 5 (yes, more than 10 times).

***Peer victimization and offending*** is a scale developed by Kerr and Stattin and used elsewhere [47,48]. It assesses (1) being sexually, emotionally, physically, and/or ethnically harassed (i.e., victimization) with nine items (e.g., “Has anyone made fun of you in a derogatory way because you come from another country?”) and (2) perpetuation of harassment and offending with five items (e.g., “Have you beaten, kicked, or assaulted anyone in an unpleasant way at school or on the way to or from school?”)

***Delinquency*** developed by Swedish National Council for Crime Prevention [49], this measure assesses adolescent engagement in delinquent behaviors with 13 items (e.g., “How many times have you threatened someone to get that person’s money or other belongings?”) rated from 1 (never) to 3 (several times).

***Substance use*** includes both questions used in the Swedish Council for Information on Alcohol and Other Drugs annual survey on adolescents’ alcohol and drug use [50] on the extent of use of alcohol and narcotics, as well as the Alcohol Use Disorders Identification Test and the Drug Use Disorders Identification Test [51] used for screening of alcohol and drug abuse.

Promotive mechanisms are operationalized through the following measures:

***Student perseverance and self-efficacy***, as assessed by teacher self-reports, uses six items to measure the extent to which the student maintains engagement with schoolwork (e.g., “Does the student show perseverance with different school assignments?”) rated from 1 (not at all) to 5 (very much).

Academic achievement is operationalized through the following measures:

***School achievement*** (grades), merit points, high school eligibility, and absence from school are collected from school records and national register data [45].

Mental health and wellbeing are operationalized through the following measures:

***Psychosomatic problems*** [52] is measured with the Health Behaviour in School-aged Children which assesses, with eight items, the extent to which adolescents experience psychosomatic problems such as sleep issues, stomachache, and worries.

***Perceived disability and support*** is assessed both by adolescent self-reports (e.g., “Do you have attention deficit hyperactivity disorder, Autism, learning disabilities?”) and (e.g., “Have you been in contact with school health care?”) and teacher reports on their observed student disabilities and support (e.g., “Does the student have a reading disability?”) and (e.g., “Does the student receive any accommodations or modifications at school?”)

***Psychiatric symptoms*** are measured by the Structured Mini International Neuropsychiatric Interview [53] which assesses psychiatric symptoms according to Diagnostic and Statistical Manual of Mental Disorders (Fourth Edition [54],) and the International Classification of Diseases (Tenth edition [55], e.g., depression, suicidal thoughts, panic attacks, eating disorders).

***Wellbeing*** is measured by Mental Health Continuum – Short Form [56] which with 14 items assessing adolescent emotional, social, and psychological wellbeing (e.g., How often during the last 30 days have you felt joy?) rated from 1 (never) to 6 (every day).

Educational, employment, and income outcomes are operationalized through the following measures:

***Education, employment, and income.*** Final grades from upper secondary school, data on youths who are Not in Education, Employment or Training (NEET), ongoing education, employment or internships, and income and source are collected from the national registers, such as Sweden Statistics and Swedish Social Insurance Agency. The occurrence of economic assistance was obtained from the register of economic assistance at the National Board of Health and Welfare.

#### Quantitative data analyses.

To comprehensively understand the longitudinal processes and to capture the dynamic nature of development and the interactions between variables over time, advanced longitudinal statistical analyses are essential. Specifically, we will employ structural equation modelling (SEM) to identify and test the relationships between variables within our conceptual framework. The SEM analyses allow us to control for variation in outcome variables over time. Additional covariates (e.g., socioeconomic status, gender, ethnicity, function, and ability) will be leveraged in order to assess their impact on models. The large cohort size, repeated measurement design, and register-based follow-up provide sufficient statistical precision for the planned analyses of associations.

Given that our data is nested within schools, it is crucial to account for the hierarchical structure of the data. Therefore, multilevel analyses will be utilized to address the dependencies within the data, ensuring that the variations at the school level are appropriately considered. In addition, we will use Latent Growth Models (LGMs) to examine trajectories of change over time, allowing us to identify patterns of development and factors that influence growth rates. This is particularly important for understanding how variables evolve across different stages of adolescence and into early adulthood. Furthermore, cross-lagged panel models with interaction effects will be employed to investigate the reciprocal relationships between variables and how these interactions may vary over time. These models will help us determine the directionality of effects and identify potential mediating and moderating variables that could influence the developmental pathways of interest. This in turn can be used for targeting and issue prioritization in later intervention and policy development where relationships that have strong predictive power can be prioritized above relationships with weak predictive power.

#### Missing data, sensitivity analysis, and model selection.

Missing data will be handled using full information maximum likelihood (FIML) estimation within the structural equation modeling framework. FIML uses all available information from the observed data under the assumption that data are missing at random (MAR) and is widely recommended for longitudinal SEM and latent growth models. To support the MAR assumption and reduce potential bias, we will include relevant auxiliary variables (e.g., baseline characteristics and prior measurements related to attrition and nonresponse). As a sensitivity analysis, we will compare key results with models estimated using multiple imputation to evaluate the robustness of our findings to different missing data handling strategies.

Model selection and evaluation will follow established SEM guidelines. We will assess global model fit using the χ² test, RMSEA (with 90% CI), SRMR, CFI, and TLI. Competing models (e.g., alternative growth forms or cross-lagged structures) will be compared using AIC, BIC, and sample-size adjusted BIC, with preference given to more parsimonious models when fit is comparable. Parameter estimates will be evaluated for statistical significance, effect size, and theoretical interpretability. Measurement invariance across time will be tested to ensure that longitudinal comparisons are meaningful. Model modifications will be considered only when theoretically justified.

To ensure robustness and reproducibility of our findings, we will (a) evaluate alternative model specifications (e.g., different growth forms and cross-lagged structures) and compare results across these models, (b) conduct sensitivity analyses examining the impact of covariate inclusion and different missing data handling approaches (e.g., FIML and, where appropriate, multiple imputation), and (c) test measurement invariance across time to confirm that observed changes reflect true change in the constructs. We will use robust estimators and report full model details, including syntax, fit indices, and decision criteria, in supplementary materials to facilitate independent replication

### Group interviews and workshops with teachers and students

To answer RQ3 and RQ4 and to understand how school staff and students experience their school environments and possibilities for positive and healthy relationships, learning and achievement, we will conduct group interviews [57] with school staff and workshops [58] with students and school staff at two public middle schools. Selection of middle schools will be based on convenience and willing ness to participate in the project. In addition, selection will be limited to large middle schools (> 100 students per grade) with diverse student bodies. These methods will include questions about the perceived positive and negative aspects of the school environment, including learning spaces and break time activities, organizational values and climate, interactions among students and teachers, and the perceived support provided and received. They will also address the challenges that students and school staff encounter in their daily school life, how difficult situations are handled, and the general and specific needs of students and staff to improve the school both as a learning environment and in terms of health opportunities.

#### Qualitative data analysis.

Qualitative data analysis will be conducted using reflexive thematic analysis [59] which includes descriptions of different patterns, or themes provided in the data. As suggested by Braun and Clarke [59] we will begin with structural coding after the data has been transcribed. These codes will identify features of the data that are of interest for the objectives of the study. When the whole data set has been coded, we will start analyzing the codes, considering how different codes could be combined to an overarching theme. In the next step, we will refine the themes in the data. In the final step we aim to be able to identify the essence of each theme and finally define what those themes are.

### Co-development and pilot-evaluation of school intervention

RQ5 is answered via a formative research process. The results from RQ1–4 will provide a basis for development of a school-based intervention following the four-step guidance for research co-production [60] and a partnership approach as described by O’Cathain and colleagues [61] and Croot and colleagues [62]. This co-produced intervention will contribute to increased knowledge on how effective preventive and health-promoting interventions within various target areas of public health policy can be designed and implemented. By embedding the intervention within the existing practices and structures of the school, it aims to be sustainable, allowing schools to continue implementing the ideas even after the project concludes and research support is withdrawn. Indeed, our previous research demonstrates that both adapted and novel interventions, when tailored to fit the cultural norms and needs of the target settings, are significantly more effective in achieving desired outcomes [63]. Therefore, RQ5 will be answered via the co-production process by consensus through a series of regular development team meetings. As such, this part of the project employs an evidence-based approach to intervention development by leveraging [1] current best research evidence regarding the role of school as a determinant of health and healthy development (RQs1-2), [2] professional expertise (RQ3 and RQ5), and [3] student preferences (RQ4).

The intervention will be developed through a structured co-production process involving a multidisciplinary development team consisting of researchers and people from participating schools (e.g., school leaders, teachers, student health professionals, and students). The inclusion of both professional and student perspectives is intended to ensure that the intervention is evidence-informed, contextually relevant, feasible to implement, and responsive to the needs and experiences of those who will use it.

Intervention development will be informed by findings from RQ1–RQ4. Specifically, results from the longitudinal analyses will be used to identify school-level determinants that are associated with student achievement, wellbeing, and health outcomes, while findings from the qualitative studies will be used to identify perceived needs, barriers, resources, and opportunities for change. Through a series of co-production workshops, the development team will collaboratively identify intervention priorities, develop intervention components, and refine the intervention prior to pilot testing.

All meetings and group discussions will be recorded as a means of data collection. These recordings capture the rich dialogue and exchange of ideas among project team members, stakeholders, and experts. By analyzing these recordings, we will gain insights into the critical components of the intervention, such as key themes, emerging patterns, and areas of consensus or divergence. Furthermore, the recordings will provide a record of the delivery of intervention outputs, allowing for retrospective analysis and refinement of intervention strategies as needed. Overall, this comprehensive approach to data collection ensures that interventions are informed by multiple sources of evidence and stakeholder input, thereby increasing relevance, effectiveness, and sustainability. Data will be analyzed with reflexive thematic analysis [59] and qualitative content analysis (e.g., [64]). Work within this RQ will lead to three concrete deliverables: An intervention theory of change (e.g., logic model; [65]); an intervention manual (e.g., [66]); and a teacher training package (e.g., [67]).

Although the exact intervention content will be determined through the co-production process, it is anticipated that the intervention will target modifiable dimensions of school climate, including school connectedness, teacher-student relationships, peer relationships, and supportive school environments.

To answer RQ6 and to evaluate the initial impact of the school intervention on school climate and student health behaviors we will conduct a small scale pre- and post-plot test with approximately *N* = 30 teachers and other school staff, including school health care, and *N* = 150 students from 7th to 9th grade. For the pilot, we will assess both staff-focused and student reported relational aspects of school climate. Staff-focused indicators will include job satisfaction, organizational climate, and organizational values which are early indicators or change. Relational climate will be captured through student reports (e.g., [68]). In this pilot phase, these variables will be used primarily to examine feasibility of measurement, variability, and sensitivity to change, providing preliminary signals to guide refinement of the intervention and selection of primary outcomes for a subsequent full-scale study. We will also collect registry data on grades and absence from school pre- and post-intervention. It is possible that other measures will be used based upon the intervention developed and its specific theory of change. We allow for this based on the co-production aspirations and, as such, it is important that school staff are involved in selecting final measures. Post-intervention data collection will be planned in relation to final intervention characteristics (e.g., length and timing within the school year).

Given the complexity of school climate and other organizational processes, substantial changes may take time to emerge. Accordingly, the primary purpose of the pilot study is to assess feasibility, acceptability, implementation, and preliminary indications of change rather than to establish definitive intervention effectiveness. Findings will be used to refine the intervention and inform the design of future larger-scale evaluations with longer follow-up periods.

Repeated measures analysis of variance will be used to compare data between the time points. For the student pre-post analyses, a sample of 150 students provides adequate precision to detect small-to-moderate within-person changes, while the staff sample of 30 provides preliminary estimates of change and implementation feasibility. Findings from the pilot will be interpreted cautiously and used primarily to refine the intervention and inform sample size calculations for a subsequent adequately powered evaluation.

Finally, to answer RQ7 and understand the feasibility of the intervention, we will conduct focus group interviews with students and teachers respectively. With these interviews we want to investigate perceived barriers and facilitators to implementing the school-based intervention. Specifically, the following themes will be explored: acceptability of the intervention (e.g., satisfaction), feasibility of the intervention, perceived appropriateness of the intervention, fit of the intervention within the organizational culture, perceived success/failure with the intervention, amount and type of resources necessary to implement the intervention, factors affecting implementation ease or difficulty, perceived quality of implementation, perceived effects (positive/negative) on participants, perceived sustainability, and perceived fit with organizational goals and culture. These measures are often considered “leading indicators” of implementation success [69].

As the intervention is under development, specific fidelity and implementation measures will not be finalized within the context of this project. However, we will follow established implementation frameworks and assess key dimensions of fidelity, including adherence to core components, dosage (exposure), quality of delivery, and participant responsiveness retrospectively as part of the posttest interviews in order to aid in development of specific measures and processes within the development framework.

### Ethical considerations and declarations

Principles of ethics in research are the primary guiding principles of this project. The ethical framework guiding this study includes procedures for consent, confidentiality, recruitment, and participant protection, all of which comply with Swedish ethical review regulations. All study procedures are informed by these ethical standards, as well as by best practices in research according to Swedish law and the guidelines of the Swedish Research Council. The Principal Investigator (PI) has overarching responsibility to ensure compliance with these standards. Ethics approval has already been obtained (Regional Research Review Board in Gothenburg No. 362−13, 25 September 2013, 20 May 2014, 2 September 2015, 25 July 2017, 26 July 2018; Swedish Ethics Review Authority No. 2019−00664 29 January 2019, 2021−01443, 8 April 2021, 2024-03620-02; No. 2025-05596-01 12 November 2025).

For research questions 1–2 we do not collect any primary data. Instead, we collect data from a large Swedish longitudinal study which is housed at Jönköping University, Jönköping Sweden. Details about the longitudinal study and the procedures for access to the data as well as participant consent, recruitment, protection procedures and other ethical considerations have been extensively documented previously [45]. This data is pseudo-anonymized, and we have no access to identifying information on the young people who make up the GMC. For research questions 3–7, school staff receive oral and written information during staff meetings. Staff who are absent receive the information via e-mail. During data collection, staff are reminded of the study procedures and their right to opt out. As data are generated at the group level and no personal information is collected, researchers cannot identify who has participated; therefore, individual staff consent is not documented. Guardians receive written information via the schools’ digital platform, in both Swedish and English. Guardians provide informed consent through a digital consent form on the platform. Information posted on the platform is followed up by contacting guardians via e-mail or telephone. When contacted by e-mail, a link to the electronic consent form is provided; when contacted by telephone, oral information is given and oral consent is requested. The child’s name and personal identity number, as well as the guardian’s name and contact details, are documented together with the consent. Students whose guardians provide informed consent receive written information via the schools’ digital platform and oral information from their mentors. Mentors are provided with video clips explaining the study, and the research team also provides oral information during school visits. Students are asked for assent on each occasion and may opt out of data collection at any time. They are informed that they do not have to submit the survey and may skip any questions they do not wish to answer.

This project involves participants under the age of 18, who are considered vulnerable due to their status as children. When working with child participants, researchers must exercise extra caution. This includes ensuring that information about the study is clear and understandable, enabling informed decision-making. Informed consent will be solicited from all participants as well as parents of children by the research team. Additionally, instruments need to be adapted appropriately for the target age group. There is a risk that children may feel uncomfortable answering certain questions or that the behaviors being asked about could be exacerbated, known as the question-behavior effect. However, research findings on this effect are inconsistent (see, e.g., [70] for research on suicide). We will follow established guidelines throughout the project, especially during interviews. Moreover, participants will be informed about whom to contact at their school (e.g., school nurse) if they need support. All collected and stored data will be deidentified, and this project does not aim to collect primary data of a sensitive nature as defined by Swedish law. Despite some risks, particularly for children, empirical research is crucial for designing and delivering effective school-based interventions. We believe that participating students and school staff will find their involvement positive, as it provides them with an opportunity to be heard and feel that their experiences and opinions are valued. We will actively monitor any iatrogenic effects of the intervention, and if indications of harm arise, we will strongly recommend discontinuing its use. The research aims to support knowledge-based practices in participating organizations. The potential benefits of the research, along with measures to mitigate harm, lead us to conclude that the benefits of the research program outweigh the risks, making the research ethically justifiable.

#### Timeline of the study.

The study timeline can be found in Fig 2.

**Fig 2:**
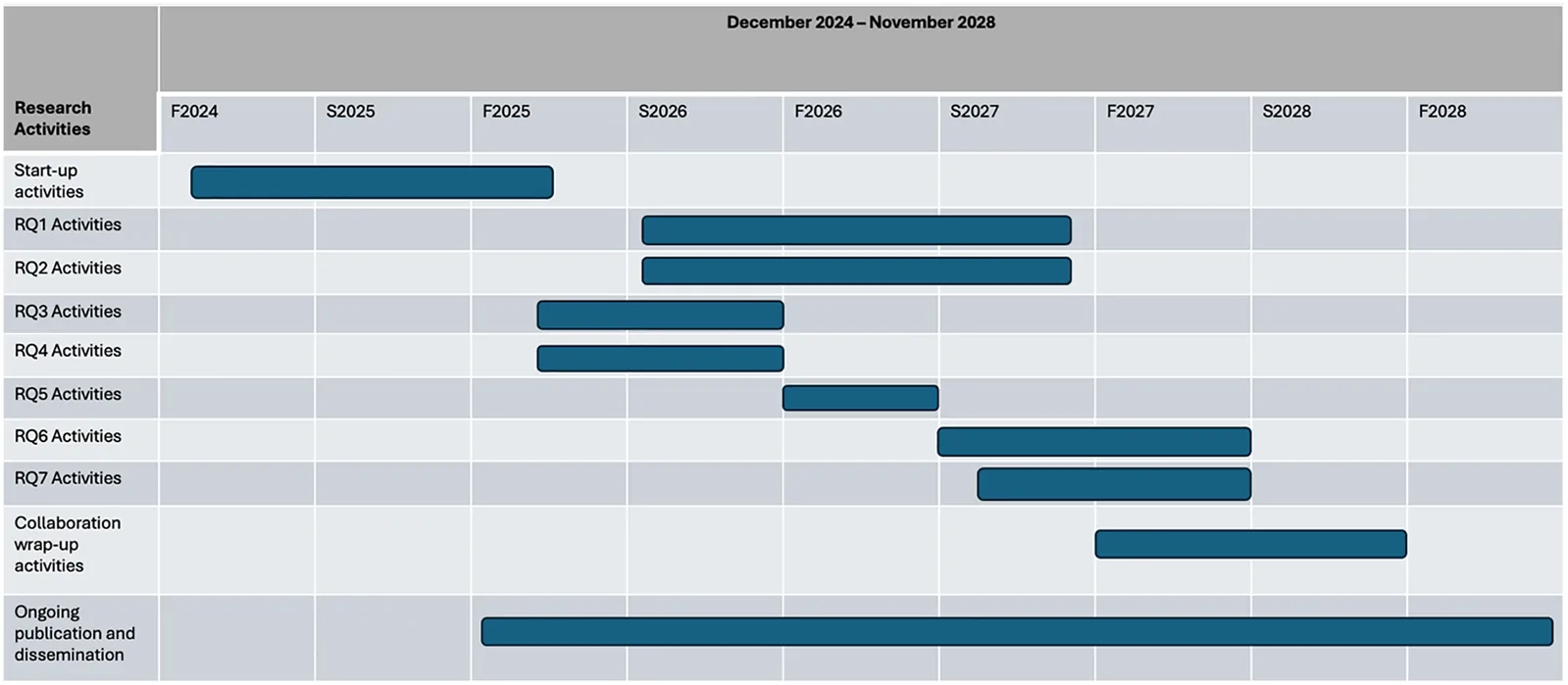
Study time-line.

## Discussion

This project aims to offer significant societal benefits by understanding and improving factors that influence students’ health behaviors and academic achievement thus fostering a healthier, more equitable society in the short- and long-term. The findings can inform policies and practices that enhance individual and community well-being, leading to societal improvements. One of the primary societal utilities of this research is its potential to enhance educational outcomes. Educational attainment is a fundamental SDOH, influencing individuals’ opportunities for employment and income. By identifying and addressing school-level SDOH, such as student behaviors, school climate, including the quality of peer and student-teacher relationships, this research can help create educational environments that support all students’ success. Improved educational outcomes are likely to lead to better job prospects and higher incomes, reducing socioeconomic disparities. By examining how different groups of students, including those from marginalized backgrounds, are affected by school-level SDOH, the project can identify and address disparities. This commitment to equity ensures that all students have the opportunity to succeed, regardless of their background. The consequences of low academic achievement are intergenerational [71] and as such, increasing our understanding of how the school arena may work to positively impact educational achievement has consequences for the intergenerational transmission of inequality in employment, income, and health.

The project can also significantly impact public health by promoting healthier behaviors and environments in schools, reducing health issues among young people. Improved school climate that includes positive interactions can lead to healthier lifestyles, reducing the burden of chronic diseases and mental health issues, and easing pressure on healthcare systems. Additionally, developing and piloting interventions offers direct societal value by providing evidence-based strategies for educators, policymakers, and community leaders. These interventions can be scaled and implemented widely, leading to broad improvements in student health and achievement. The knowledge generated can inform future research and innovation in education and public health, fostering continuous improvement.

Developing people’s skills and knowledge through learning and education is central to achieving equitable health [5]. There is a strong correlation between education level and health. Acquired knowledge and skills through education provide opportunities for better jobs and higher wages and strengthen psychological and social resources and real opportunities to influence one’s situation, which can reduce physical and psychosocial risks. Living conditions, lifestyle habits, and other factors contributing to good health are better and more health-promoting among individuals with higher levels of education. Therefore, it is important to develop cognitive and non-cognitive abilities and for schools to contribute to all children’s learning and development, regardless of individual circumstances and parents’ background and level of education. Research suggests that school-level factors can adversely impact student achievement and attainment on equal grounds. As such, this project is relevant to society on many levels. First, this project is relevant to children and families as it aims to understand and impact the school environment for children by developing an understanding of how school climate impacts student health and learning in the short term and contributes to employment, wages, and health in the long term.

The project is also of relevance to school staff (e.g., teachers, leadership). Staff within schools are a group that stands to benefit from this project as school climate has been found to be significantly related to teacher well-being and turnover [72], which has spillover effects on student learning and achievement [73]. More distal societal relevance is related to the impact on communities that arise from school drop-out and low achievement above and beyond that of employment, income, and health outcomes [74] for individuals, such as their impact on neighborhood crime [75,76] including violent crime, property crime, arrest rates, and drug use [77]. On the macro level, this project is relevant to broader societal and policy/political interests as relates to productivity, through impacts on education, employment, and health through a greater understanding of how we might build better schools for equitable health promotion.
